# Stem Life: A Framework for Understanding the Prebiotic-Biotic Transition

**DOI:** 10.1007/s00239-024-10201-z

**Published:** 2024-09-08

**Authors:** Gregory P. Fournier

**Affiliations:** Department of Earth, Atmospheric, and Planetary Sciences, Massachusetts Institute of Technology, Cambridge, MA USA

**Keywords:** Prebiotic chemistry, LUCA, Cladistics, Evolution

## Abstract

Abiogenesis is frequently envisioned as a linear, ladder-like progression of increasingly complex chemical systems, eventually leading to the ancestors of extant cellular life. This “pre-cladistics” view is in stark contrast to the well-accepted principles of organismal evolutionary biology, as informed by paleontology and phylogenetics. Applying this perspective to origins, I explore the paradigm of “Stem Life,” which embeds abiogenesis within a broader continuity of diversification and extinction of both hereditary lineages and chemical systems. In this new paradigm, extant life’s ancestral lineage emerged alongside and was dependent upon many other complex prebiotic chemical systems, as part of a diverse and fecund prebiosphere. Drawing from several natural history analogies, I show how this shift in perspective enriches our understanding of Origins and directly informs debates on defining Life, the emergence of the Last Universal Common Ancestor (LUCA), and the implications of prebiotic chemical experiments.

## Introduction

**Fig. 1:**
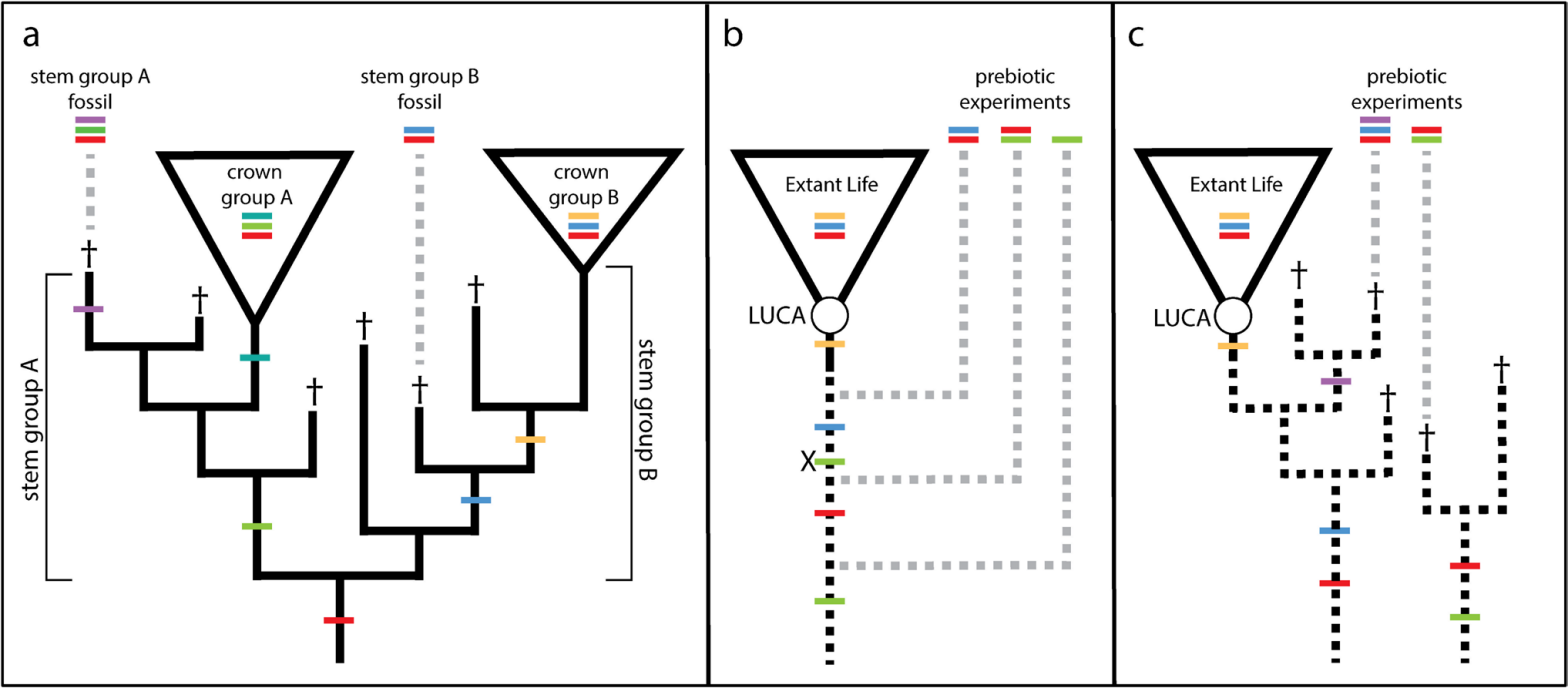
Reconstructing stem groups within lineages. **a** Fossils as sampled representatives of extinct stem groups possessing a subset of derived characters found within crown groups. **b** Prebiotic chemistry experiments as sampled transitional forms from a pre-cladistic “ladderized” origins narrative. **c** Prebiotic chemistry experiments as sampled hypothetical states of extinct stem life possessing subsets of properties shared with extant life. Daggers represent extinct stem groups. Terminal character states/acquisitions are encoded by colored bands, with “X” in panel b denoting a character loss. Vertical axis (time) is not scaled

One of the most important concepts in cladistics is that of the “stem group,” the extinct diversity of life that falls outside of crown group clades observed today (Budd and Jensen 2000; Dupuis 1984; Hennig 1966; Richter and Meier 1994). Stem groups are included within a “total group” defined as all extinct and extant organisms more closely related to one another than a sibling clade; however, they may share only some or even none of the derived characters acquired by the lineage leading to the crown group (Fig. 1a). This extinct diversity is unknowable except in the form of the fossil record, but can always be inferred to have existed, given the universal pattern of phylogenesis in Darwinian evolution: a Tree of Life shaped by continual processes of speciation and extinction (Barton et al. 2007). While well established in organismal evolutionary biology and paleontology, these concepts have yet to be fully applied to our thinking about origins, namely, the transition from prebiotic chemistry to Life, the origins of the first cellular lineages, and the emergence of the Last Universal Common Ancestor (LUCA) and its descendants. The series of transitions inferred to have occurred from prebiotic synthesis to complex autocatalytic chemical systems and eventually to cellular life is still generally conceptualized in a pre-cladistics framework—one of a linear, stepwise progression of forms, with each “higher” form displacing the previous “lower” form, and each intermediate a necessary step in the emergence of life as we know it (Fig. 1b). This perspective is not only inconsistent with the observed patterns of biological evolution today, but leads to an overly stringent and limited framework of inquiry in prebiotic chemistry studies.

One of the most universal aspects of nature is *fecundity*—the ateleological cycling of matter and energy into countless forms. The story of life is, for the most part, the story of evolutionary dead ends; most species have left no extant descendants or evidence they ever existed. Buried within this astounding gravidity are slender threads of lineage—histories of genes, cells, and organisms that can be traced back to common ancestors. Tracing these lineages through comparative genomics and phylogenetics is one of the most powerful tools of modern biology. However, it can also mislead our thinking: reconstructing the history of life only by working backward from extant life provides an impoverished and inaccurate view of the life and ecology of the past, the so-called “Pull of the Recent” (Raup 1972). As one moves backward along a phylogenetic tree generated from extant taxa, the lineages traversed represent an ever-decreasing proportion of the life extant at that time. As such, they are also decreasingly likely to be representative or ecologically relevant. For example, a phylogenetic reconstruction of amniote evolution from extant representatives would accurately recover the relatively recent Cenozoic radiations of Aves and Mammalia, but completely fail to discover the ecologically-dominant terrestrial fauna of the preceding periods, including major groups of non-avian archosaurs in the Mesozoic (Brusatte et al. 2010), parareptiles in the Permian (Tsuji and Müller 2009), and non-mammalian synapsids in the Permian and Triassic (Kemp 2006).

The ecological significance of precursor lineages should also not be evaluated in terms of their future persistence or extinction, as the latter can have no bearing on the former. Such an ahistorical lens threatens to limit our understanding to a likely misleading “presentism,” where some groups are apparently predestined for greatness as a virtue of their ancestral traits. The interdependency of organisms in the ecologies of the past makes no distinction with respect to the future persistence or eventual extinction of their constituent lineages. This is clearly true in the history of complex life (e.g., Paleozoic reefs formed by stromatoporoid sponges (Kershaw 1998) and rugose and tabulate corals (Drake et al. 2020)) and is even likely the case in microbial evolution; for example, life in Archean oceans may have been largely sustained by ancient groups of photoferrotrophic bacteria with modern analogs but no surviving descendants (Stuekeen et al. 2020). In the case of microbial lineages, traits themselves often have complex, reticulate histories, with genes and their associated phenotypes spread via horizontal gene transfer (HGT) between lineages. Nevertheless, cladistics still traces the acquisition and presence of these traits within lineages, independent of the processes by which they were acquired. That being said, the larger role of HGT within microbial lineages favors this process for the persistence of traits independent of lineage histories, which, in complex organisms, is largely a result of convergent evolution. For example, modern photoferrotrophic bacteria (Gupta et al. 2021; Camacho et al. 2017), while not descendants of their extinct Archaean forerunners, almost certainly use the same photosynthetic machinery, acquired via a long history of intermediary HGTs.

While the implications of stem groups are well understood in modern evolutionary biology and paleontology, these are rarely applied to microbial evolutionary history and even more rarely, if ever, applied to questions about the Origin of Life. The Pull of the Recent can therefore limit our thinking about Origins in an even more extreme way, due to a total lack of paleontological evidence to push back against such bias. Extending the cladistics concept of the stem group to encompass the history of life before LUCA can help in avoiding these biases and aid in developing a conceptual framework for understanding early life and prebiotic systems in a more holistic and uniformitarian way. Therefore, I propose characterizing the entire diversity of biotic and prebiotic systems diverging before LUCA as “Stem Life.” This framework can also aid in our interpretation of the results of prebiotic chemistry experiments, freeing them from the overly stringent and untestable hypothesis that each reconstructed prebiotic system, if “true” must therefore represent a necessary step in the direct chain of events leading to life as we know it. Rather, these experiments should be considered as attempts at reconstructed samplings of diverse chemical systems that are the products of a fecund and interconnected prebiotic world, possibly related to, but not necessarily ancestral to, extant life (Fig. 1C).

### Definitions of Life and Apomorphy-Based Cladistics

The term Stem Life is especially useful in that it avoids assuming or requiring any definition of life in order to separate the prebiotic and biotic worlds. Definitions of life are notoriously problematic for either being inclusive of obviously non-living systems, potentially exclusive of pre-LUCA or alien life, or inherently lacking in utility (Benner 2010; Szostak 2012; Machery 2012). It is likely that any definition is subjective and imperfect and so “working definitions” that are primarily evaluated by their usefulness in generating testable hypotheses are preferred, such as the NASA working definition of life: *“Life is a self-sustaining chemical system capable of Darwinian evolution”* (Joyce 1994). Interestingly, modern systematics and paleontology often face analogous dilemmas when defining membership within a taxonomic unit, and considering these cases can guide our thinking about challenges in defining life.

The strictest definition of a natural taxonomic grouping is that of the crown group (clade). This definition is purely objective, relying only upon the topology of the inferred phylogenetic tree. Such a definition excludes closely related stem groups that may possess most or all of the characters (apomorphies) of the crown group, including apomorphies that are physiologically or anatomically unifying. For example, the strict, crown group definition of the class “Aves” includes the last common ancestor of all extant bird species and all of its descendants (formally, Neornithes) (Gadow, 1893). However, this definition is sometimes considered overly strict, as it excludes stem bird lineages that possessed *“feathered wings used in flapping flight”* (Ostrom et al. 2001), which would clearly meet the common notion of what a bird is, as opposed to all other theropod dinosaurs. Therefore, an apomorphy-based clade definition inclusive of these close relatives is sometimes used for Aves, “Avialae” (Ostrom et al. 2001). Such an apomorphy-based definition is nevertheless inherently subjective, and alternative schemes based on other apomorphies can also be proposed. In the case above, Avialae would include representatives with saurian-like tails (e.g., *Archaeopteryx*) and thus not fully resemble the body plan of modern birds. In contrast, a more restrictive apomorphy-based grouping for Aves has also been proposed, “Ornithurae,” which only additionally includes stem birds with fused pygostyle tails similar to modern birds, a more recently evolved trait (Haeckel 1866).

Both crown- and apomorphy-based group definitions are enabled by the apparent discontinuity of the observed diversity of extant and past life, because extinction and the sparseness of the fossil record have masked the continuity of forms that have existed in the past. The same is almost certainly true for the diversity of prebiotic and biotic forms preceding LUCA: a continuity that, if observed in its completeness, would defy any objective definition of “Life.” In the strictest possible view, we could apply a crown-based definition of life that life is simply defined as LUCA and all of its extinct and extant descendants. But this is a trivial and unsatisfying definition, and does not provide any predictive power in understanding Origins, or recognizing other forms of life elsewhere in the universe. Furthermore, it would exclude any extinct lineages that diverged before LUCA, even if they possessed all of the cellular machinery and processes shared by extant life (Cantine and Fournier 2018; Fournier et al. 2015). Defining the origin of life as coincident with LUCA inherently elevates LUCA to a special evolutionary status without justification; the coalescence of all organismal lineages to a single ancestor is an inevitable consequence of cladogenesis and does not, in itself, indicate any notable event occurring at this point in evolutionary history. Any meaningful and potentially useful definition of life is therefore, in effect, a proposed apomorphy-based definition that is not only met by the crown group of Life, but by other more distantly related forms as well. However, any defining apomorphy, be it cellularity or nucleic acid heredity, also immediately excludes the closest related forms as “not life” or “almost life.” It is therefore difficult to see how to avoid a slippery slope of definitions, especially in the absence of any empirical evidence about the order in which different properties inherited by extant life emerged.

As a concept, Stem Life does not depend upon any apomorphy or definition of life to be applied and thus transcends these difficulties. This is one of the key utilities of stem groups in general; they can encompass a wide range of evolutionary hypotheses in the absence of fossil evidence. Stem Life reflects a similarly appropriate agnosticism about the evolutionary transitions and forms that preceded LUCA.

### The Problem of the Outgroup

Applying the concept of the stem group to pre-LUCA divergences faces one unique challenge: there is no outgroup. By definition, LUCA is the “event horizon” of comparative phylogenomics. The evolutionary histories of some gene families can be traced deeper than LUCA, with pre-LUCA divergences representing gene duplication events (Iwabe et al. 1989; Brown and Doolittle 1995; Gribaldo and Cammarano 1998), but these divergences do not resolve any organismal lineages before the divergence of Archaea and Bacteria at the point of LUCA. As such, in this case the definition of a stem group as “all lineages more closely related to the crown group than the outgroup” appears to be nonsensical. Is an outgroup necessary to the concept of a stem group? If we consider the case of an unresolved phylogeny where the outgroup is not known, a stem group can still be inferred, if extinct lineages are shown to place outside the crown group. For example, stem group turtles have been identified and described (Schoch and Sues 2020), even in the absence of a reliable placement of Testudines within the amniote tree (Field et al. 2014; Lyson et al. 2012). However, even in these circumstances, comparisons are still being made to other lineages, which define the synapomorphies by which the stem groups may be placed. If that is the utility of the outgroup, then we can safely discard it with respect to pre-LUCA divergences, since there are no alternative hypotheses to evaluate; we are not testing whether pre-LUCA divergences are truly as such, but are merely using cladistic nomenclature to describe them in a useful manner.

### Ancestry vs. Heredity

Another problem to be considered is what is meant by “ancestry.” Normally, this is not a problem in cladistics, as it is taken for granted that every speciation event involves heredity, that is, the passing down of genetic information via genome replication and cell divisions. But at some point before LUCA, tracing the ancestry of life may push beyond the origins of cells and nucleic acid genomes and conventional notions of heredity. This is distinct from the hypothesized “Darwinian threshold” or transition from a progenote, which, in any of its formulations, still assumes the inheritance of genetic information (Woese 2002). Rather, this is in reference to ancestry in the purely physical sense, whatever this material may be (presumably, the components of some sort of autocatalytic chemical system that bestow continuity of function). Extending ancestry beyond heredity is similar to tracing the history of the division of cells, which is arguably little more than tracing the history of a set of lipid molecules’ collective organization. From a non-genome-centric perspective, this “history of cells” can even be considered the true history of life, with genomes merely serving as a convenient albeit imperfect tracer of cellular divisions (Doolittle and Brunet 2016; Forterre 2015). However, even this holistic view does not explicitly extend ancestry to pre-cellular chemical systems.

### “Stem Life” vs. “Pre-LUCA”

**Fig. 2:**
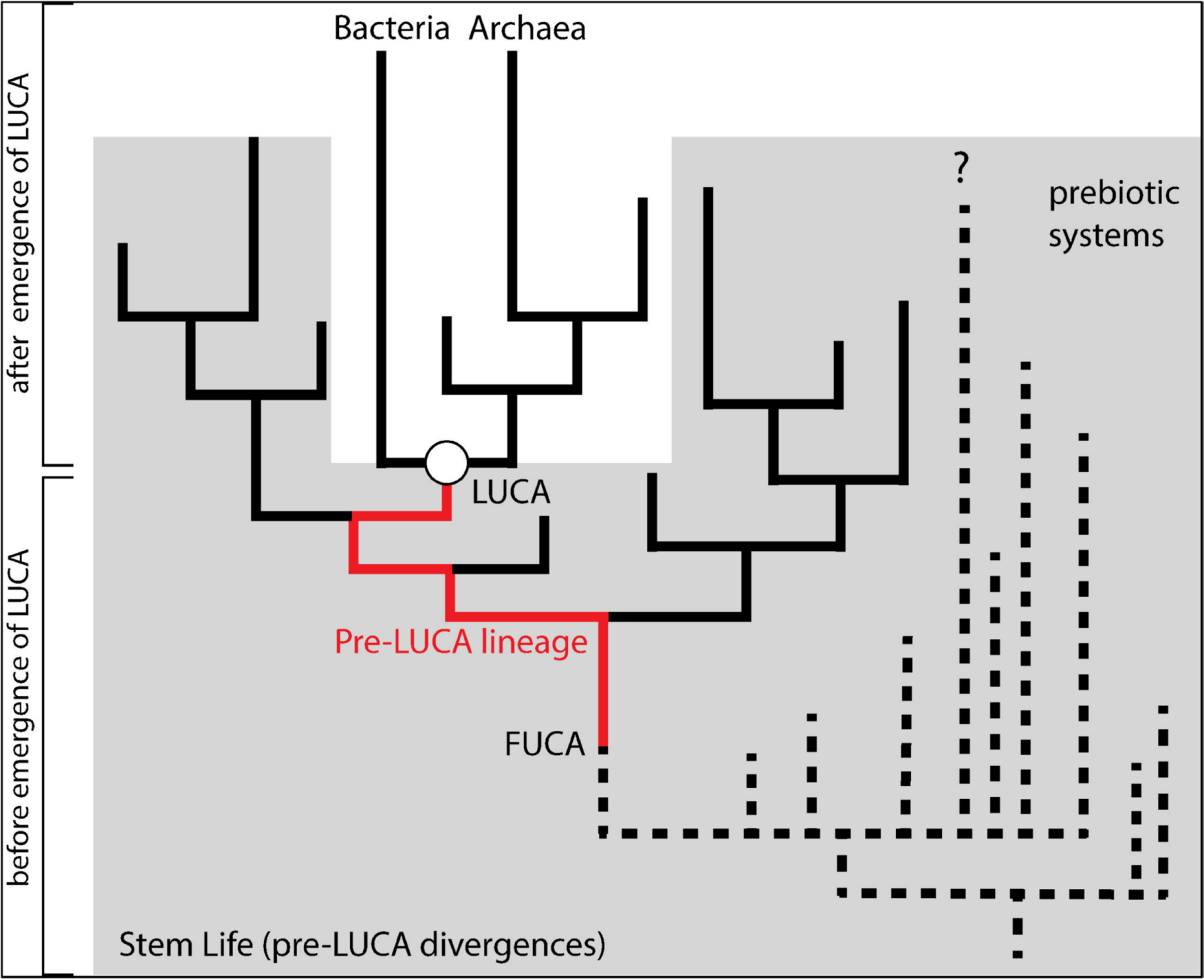
Stem Life and pre-LUCA divergences. The pre-LUCA lineage (red) shows the line of direct ancestry from FUCA (First Universal Common/Cellular ancestor) to LUCA. Stem life (gray) includes all lineages and precursors that diverge before LUCA, including any descendants persisting after the time of LUCA. Dashed lines represent “prebiotic systems” inclusive of all entities undergoing complex chemical evolution. These may or may not be related by “inheritance” via autocatalysis or nucleic acid replication and so a bifurcating tree pattern is intentionally left vague

The term “pre-LUCA” is generally used to refer to evolutionary states and/or events occurring before LUCA. The notion of “before” can be understood ancestrally or cladistically, with substantial differences in meaning. In the strictest, ancestral sense, the term refers only to the direct ancestor lineage and/or prebiotic systems giving rise to LUCA (Fig. 2). This is closest to the pre-cladistics framework, and most consistent with a linear, “ladder”-like view of origins. The definition of pre-LUCA broadens considerably when a cladistics perspective is adapted, further including any lineages that diverged before LUCA, including their descendants. These pre-LUCA lineages may very well have persisted beyond the time of LUCA and co-existed with early archaeal and bacterial groups (Fournier et al. 2015) (Fig. 2). Representatives may even exist to this day, although this is doubtful, as increasingly comprehensive sequencing efforts have yet to reveal their existence. As such, “Stem Life” is a more accurate descriptor of these groups. Such Stem Life groups likely retained some ancestral character states that reflect traits present within the direct ancestors of LUCA at the time of their divergence. They also likely acquired derived characters that are not shared with the direct ancestor lineage of LUCA or any of LUCA’s descendants.

### The Fecundity of Prebiotic Chemistry and the Age of RNA

It has long been appreciated that the Origin of Life need not be a linear process. “Cooperative schemas” have been previously proposed, wherein prebiotic systems with different properties combine to provide greater fitness and persistence. One example of this is the “double origin” theory, wherein protein-based and RNA-based prebiotic systems may independently emerge, later cooperating as a singular entity that is the ancestor of extant life (Dyson 1993). Other potentially cooperative prebiotic systems include self-assembling and replicating vesicles that may become associated with RNA or other chemistries that facilitate their growth or stability (Schrum et al. 2010; Zhou et al. 2020) or the co-evolution of RNA and DNA as a heterogeneous nucleic acid system (Bhowmik and Krishnamurthy 2019). Analogous to the interdependencies of extinct and extant lineages in past ecologies discussed above, it also seems likely that the properties and conditions enabling one type of proposed prebiotic system generally enable others as well. For example, macromolecular assemblies such as coacervates (Oparin and Synge 1957; Fox 1976) may not, in themselves, be the ancestors of extant life. But, they may have been an essential part of the “prebiosphere,” concentrating organic materials and nutrients, and favoring specific chemical interactions (Drobot et al. 2018). Similarly, abiotic polysaccharide accumulations (Li et al. 2019) could have produced a layer on surfaces that bound nutrients and minerals and provided a growth substrate for simple energy metabolisms. The examples above illustrate how non-linear “messiness” may not only be inevitable in complex prebiotic chemical systems (Walker et al. 2017), but necessary for a robust origin of life to occur.

This fecundity perspective also has implications for one of the most enduring and compelling narratives of prebiotic evolution, the RNA World: the hypothesis that RNA was both a catalytic and informational molecule before the trichotomy of DNA-RNA-protein present in extant life became established (Gilbert 1986; Higgs and Lehman 2015; Neveu et al. 2013). Preceding both DNA and genetically encoded catalytic protein synthesis, the RNA World is often invoked as a well-defined middle rung on the ladder of transitions from prebiotic to biotic systems. RNA does indeed possess remarkable catalytic potential that appears to be unrealized in extant living systems (Chen et al. 2007; Horning et al. 2019), ribonucleosides can be synthesized by plausible prebiotic mechanisms (Xu et al. 2020; Xu et al. 2017), and extant RNA systems provide top-down evidence of their fundamental role in the establishment of molecular biology (e.g., Robertson and Joyce 2012; Noller 2012). How does the concept of the RNA World fit into the Stem Life narrative?

As one moves away from a ladderized view of origins, the common understanding of an RNA World becomes less tenable. The term “RNA World” implies there was an interval in evolutionary history where RNA-based systems were central to inheritance and catalysis, as DNA and proteins did not yet exist. However, even if extant life did evolve from an RNA-based precursor system, this in no way precludes coexistence and/or interdependence with other prebiotic systems that may not have used RNA. In such a scenario, our RNA-based ancestors may have only been a trace component within a much more complex prebiotic ecology. There may also have been RNA-based prebiotic entities that were not our direct ancestors, representing alternative offshoots of prebiotic chemistry. These stem RNA-based prebiotic/biotic systems may even have persisted in some environments long after the origin and diversification of cellular life. For these reasons, a better term that captures the potential importance of RNA in Stem Life would be “Age of RNA.” Analogous to the popular characterization of the Devonian Period as the “Age of Fishes” (Young 2010), the Age of RNA describes a hypothetical period in Earth’s history where RNA had a more diverse role in biochemical/prebiochemical systems than it does today. Importantly, it does not presuppose that RNA-based entities were a necessary transitional form in the emergence of extant life or that RNA-based entities ceased to exist after this time. To further extend the analogy, while all major extant groups of fish diversified during the Devonian, the most diverse and abundant group, the Placoderms (Young 2010), went completely extinct by the end of that Period and left no descendants. The most prevalent RNA-based prebiotic/biotic systems could have shared a similar fate, with only a hint of this diversity preserved and inherited by extant biology.

### Interpreting Experimental Prebiotic Chemistry

**Fig. 3:**
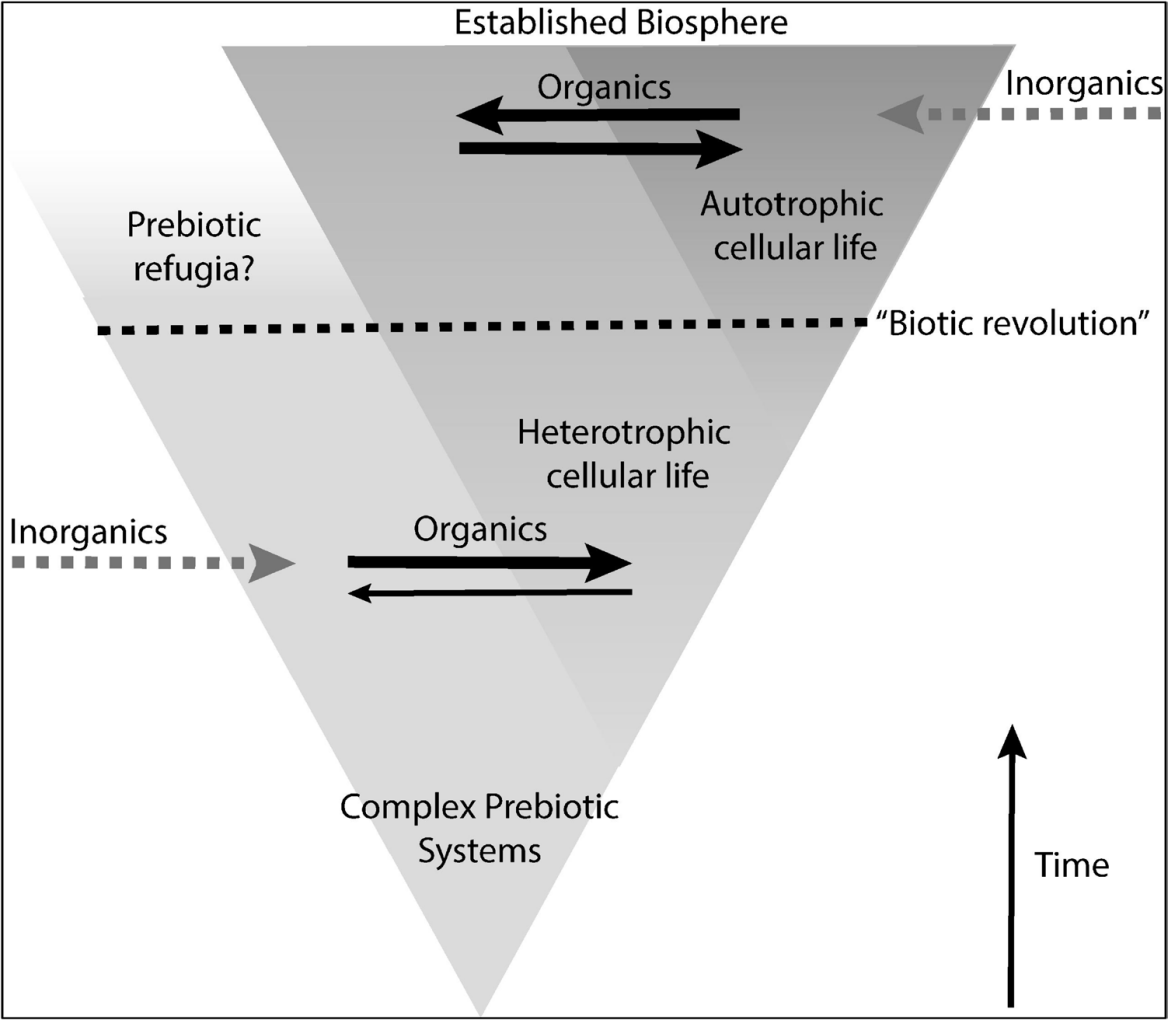
Schema for the prebiotic-biotic ecological transition. Dashed horizontal arrows represent influx of abiotically sourced compounds. Solid horizontal arrows represent flows of organic compounds within and between prebiotic and biotic systems. Arrow thickness provides a qualitative indicator of hypothesized magnitude of flow

In light of these possibilities, just what are experimental investigations into prebiotic chemistry revealing? The fossil record rarely provides “missing links” in the form of direct ancestors of extant groups, but, rather, allows evolutionary history to be *inferred* by positing relationships *between* extinct and extant forms (Fig. 1A). In much the same way, experiments designed to recapitulate prebiotic chemical systems are an attempt at sampling the solution space of potential Stem Life. Even if wildly successful, they should similarly not be understood as recovering “missing links” in abiogenesis as part of a direct ancestry to extant life (Fig. 1B), but rather as reconstructing systems that share some traits in common with these ancestors, indicative of shared ancestral states (Fig. 1C). In fact, direct ancestry is nearly impossible to prove, as alternative, unsampled forms always remain unknown, and the most parsimonious evolutionary path may not be the path that was in fact taken. Even if a given plausible reconstructed prebiotic chemical system is in fact “true”—it still may not represent the direct forerunner of living systems. It may share common origins; it may possess some shared traits, such as specific biomolecular components; it may even have co-existed with the pre-living systems that directly gave rise to life on Earth as a necessary part of their proto-ecology (Fig. 3). In this way, demonstrating the plausibility of one prebiotic system can also bolster the plausibility of broader scenarios including other systems, even if a clear link to the ancestry of extant life cannot be made. Furthermore, this perspective warns against discarding prebiotic chemical models or hypotheses that appear to lack continuity with the properties and processes observed within extant biochemistry.

### How Long Did Stem Life Persist?

The origin of modern cellular life does not necessitate or imply the erasure of all forerunner systems, no more than the evolution of tetrapods led to the extinction of all fish. In fact, even after major mass extinctions and subsequent adaptive radiations replacing dominant groups of organisms, remnant lineages of “deposed” groups often remain at the ecological margins, only going extinct much later, and with less paleontological fanfare (e.g., Jurassic and Cretaceous temnospondyl amphibians (Yates and Warren 2000)). While it is necessary that prebiotic chemistry come before the emergence of more complex biochemistry (biotic chemistry), it is not necessary that prebiotic chemical processes or even complex prebiotic chemical systems *only* existed before modern cellular life emerges or, even more strictly speaking, only existed as a direct ancestral state to cellular life. This also raises the intriguing possibility that the deepest branches of the Tree of Life reach back well into the prebiotic world. Complex prebiotic systems should persist and continue to emerge and develop as long as (1) the conditions for their synthesis persisted and (2) their products were still permitted to accumulate. Therefore, the prebiotic Earth would have ended if (1) conditions on the Early Earth were no longer conductive to the kinds of syntheses necessary for complex prebiotic chemistry to exist or (2) cellular life became successful and widespread enough to efficiently consume organic feedstocks faster than they could accumulate and undergo chemical evolution. This latter point was appreciated by Darwin himself (Peretó et al. 2009; Darwin 1887); any plausible model of abiogenesis should also be able to explain why this process apparently no longer occurs today.

This argument is not as simple as “early cells consumed all the readily available organic material.” Rather, if the prebiotic world was indeed sustained by abiotic primary production, a major transition would occur once cellular life evolved the ability to perform these reactions for itself. In such a scenario, heterotrophic cellular life, grazing on prebiotic chemical gardens and other pre-life entities, would no longer be limited to environments where complex prebiotic chemistry was sustained at high densities. This would have two consequences: (1) cellular life could disperse from its primordial environment, evolving and adapting to different environmental conditions and (2) it would become less dependent upon prebiotic chemical processes for its survival. Heterotrophic cellular life could not, on its own, be responsible for the demise of the prebiotic world, as, like in any predator–prey relationship, depletion of the food source would result in a subsequent crash in their population as well. Ironically, it may be that it was only after autotrophy evolved and became widespread that cells could use prebiotically available material to its exhaustion. This would effectively end the prebiotic world, never to return.

When would such a transition likely have occurred and what would a “transitional” world look like? One feature of prebiotic chemistry and synthesis is that it is, presumably, entirely deterministic and ahistorical; given the same conditions, the same reactions should happen again and again. This should be true across multiple locations that could experience similar chemical evolution processes, or even at the same location if a system is repeatedly extinguished, or conditions are transient. The prebiotic world in which life arose would therefore persist until some form(s) of cellular life had become sufficiently complex to disperse across multiple environments, and all abiogenic processes were globally supplanted. This would be, in effect, a “biotic revolution” and an important threshold in the establishment of a persistent biosphere (Fig. 3).

### Diversifications of Early Cellular Life

From the arguments above, we can infer that there were likely many forms of prebiotic life, co-existing and interdependent with the earliest cellular life. At this early time, diversity was primarily driven by chemical evolution, which can operate on very short, sub-geological timescales. Darwinian evolution through genetic heredity acted on by mutation and selection is a slow process by comparison, even when accelerated in the presence of high mutation rates and strong positive selection. The earliest “tree-like” diversifications of cellular life would, by definition, constitute lineages that were very closely related to one another, as so little time had elapsed since their common ancestry. In this context, vertical inheritance refers to the “tree of cell divisions,” a series of bifurcations uniting cytological and genomic histories (Doolittle and Brunet 2016). While this is only one component of the complex reticulate evolution of genomes, it is a continuous thread across all cellular lineages, traced with varying fidelity by different genes—with the most ancient, conserved genes providing the statistical proxy for the organismal (cellular) tree of life (e.g., Pugibo et al. 2013). Similar to modern organisms, early cells probably frequently recombined their genomic material, via horizontal gene transfer (HGT) with both neutral and selectively advantageous consequences. Since Woese, this has often been referred to as a “progenote” state, although the nature of the entities involved in the gene sharing is an open question (Vetsigian et al. 2006; Benner and Ellington 1990; Gogarten and Deamer 2016; Woese 1990). In such a scenario, Darwinian evolution dominated by vertical inheritance does not emerge until later, after the crossing of a “Darwinian threshold” (Woese 2002). However, an alternative scenario may be considered, by once again avoiding the biases of presentism.

**Fig. 4:**
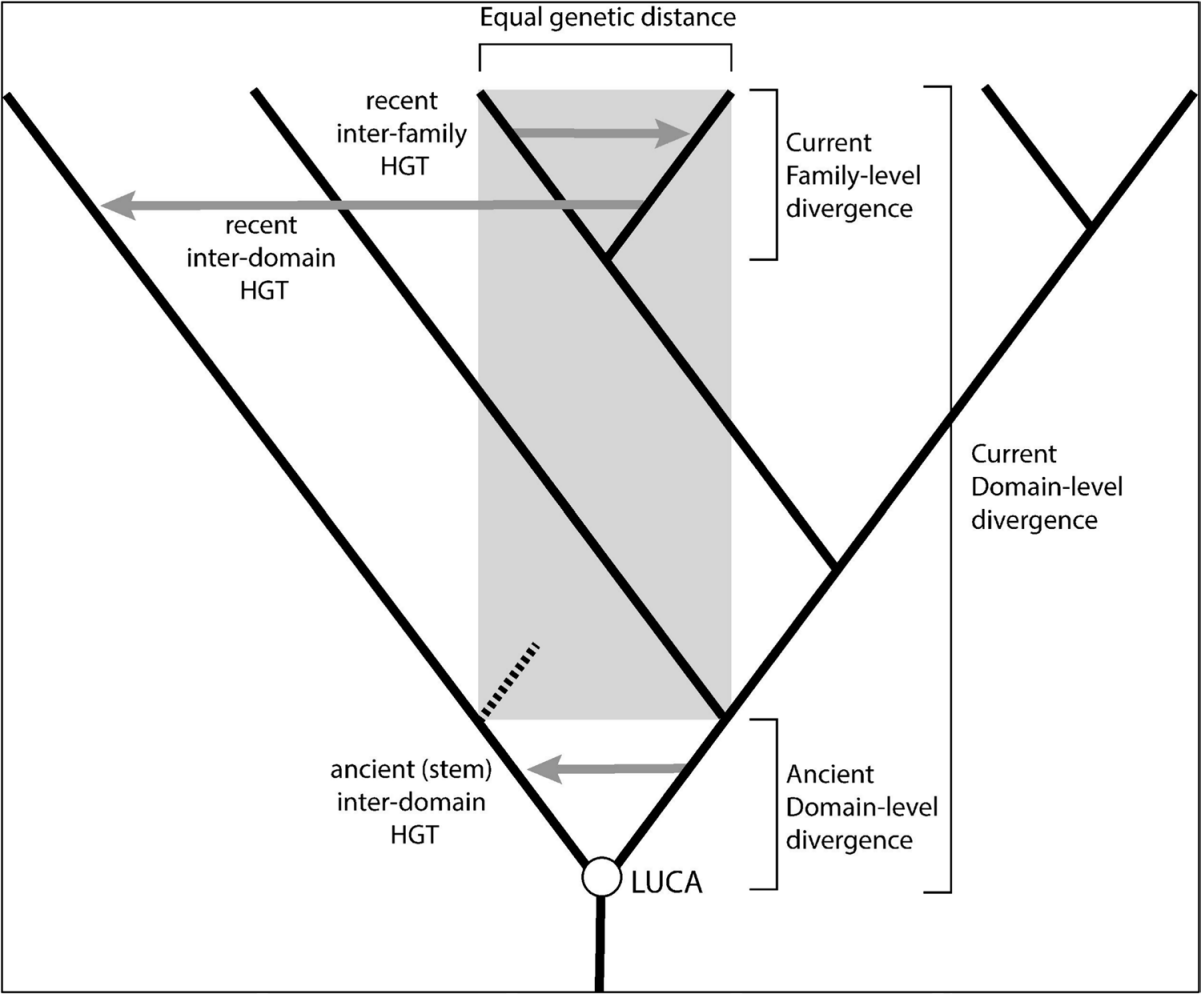
HGTs traverse greater genetic distances as the history of life unfolds. The solid line represents a simplified, bifurcating history of cellular lineages constituting the Tree of Life. Gray arrows represent HGTs, with arrow length corresponding to the relative evolutionary distance traversed. In this example, closely related groups (e.g., bacterial families) have similar sequence divergence from one another today as the entire diversity of life early in the history of the major Domains. Vertical axis of cladogram (time) is not to scale. Black-dashed line represents deeply branching lineages omitted for diagrammatic clarity

As evolution has continued over billions of years, representatives of extant lineages have become increasingly distantly related, with a longer amount of time and more evolutionary distance separating all organisms from LUCA. This may seem trivial, but the implications are profound when applied to HGT. It is well understood that closely related groups on the Tree of Life share genes at a higher frequency than distantly related groups (Kloub et al. 2021), even to the point of obscuring the signal of vertical inheritance within some clades (e.g., *Prochlorococcus* (Zhaxybayeva et al. 2009)). Gene transfers between more distantly related groups, e.g., between phyla or domains, do occur, but are observed at lower frequencies. However, the evolutionary distance that determines the expected frequency of a gene transfer is not the distance observed between the donor and recipient group today, but the *distance at the time of the transfer*, which decreases as one moves backward through time. For example, today, methanogenic archaea and cyanobacteria are separated by over 7 billion years of evolution (the sum of the time interval along both lineages of descent from their common ancestor). However, a gene transfer from methanogenic archaea to the ancestor of cyanobacteria (e.g., as inferred for the *smc* operon (Wolfe and Fournier 2018)) would have occurred over 2.5 billion years ago, at a time when the donor and recipient species were separated by around 2 billion years of evolution, with levels of sequence divergence likely equivalent to those within a single class of bacteria today. One can extrapolate even further back in time: if a few hundred million years elapsed between LUCA and the diversifications of the last common ancestors of Archaea and Bacteria, at the time their genomes would have similar levels of sequence divergence as found today within a single family of closely related bacteria, such as Enterobacteria (Fig. 4). They would be physiologically quite different, having acquired all of the characters that distinguish archaeal cells from bacterial cells today. But, if an alien exobiologist was on Earth at this time and performing environmental sequencing of ribosomal RNA genes, they would find archaeal and bacterial sequences far more similar to one another than archaeal and bacterial sequences are today. Therefore, even in the absence of some “Darwinian Threshold” or other phase change in evolutionary dynamics of genomes, it would be expected that the earliest branches of the Tree of Life would share genes at a higher frequency than what we observe within modern groups. Even though sequence similarity between lineages would have been much higher, there would also be more diversity—there would almost certainly be representative groups that branched off before LUCA or between LUCA and extant bacterial and archaeal groups that have since gone extinct. This is a pattern repeatedly seen in evolution, with “radiations” often producing short-lived periods of extreme diversity (e.g., the Cambrian Explosion (Lee et al. 2013) or the Triassic archosauriform radiation (Heckert et al. 2021; Nesbitt 2011)). Interestingly, these stem life branches would also almost certainly be sharing genes with one another, so that today’s extant lineages potentially contain a genetic testament to the existence of these “lost” primordial relatives (e.g., Fournier et al. 2015).

### Stem Life as a Copernican Solution

Each specific evolutionary transition or emergence of a particular phenotype may be, in itself, astoundingly unlikely, a unique event in the history of the universe. But, the larger the phenotypic space explored by evolutionary processes, the more likely it is that forms will emerge with any given set of traits. Many such paths are explored simultaneously and continuously, with the aid of the powerful ratchet of natural selection, which can act on chemical systems just as readily as organisms with heredity (Sharov 2016; Marakushev and Belonogova 2013). A series of specific, linear steps in chemical evolution, increasing in complexity from the synthesis of organic molecules to the origin of the first living cells, every step being both necessary and sufficient, would imply astounding luck—not only that such transitions were actualized, but that they were possible at all. However, if there are many ways to form complex prebiotic systems with life-like properties, this problem largely disappears, even if the actual path to life is only a slender, singular thread of solutions through this vast space—all that is necessary is for the system to be sufficiently fecund and persistent enough for the space to be explored. In this way, abiogenesis can be both highly contingent and still give rise, some 4 billion years later, to beings who occupy no privileged status as observers. It may still be that our existence is due to astounding chance and that the anthropic principle cannot be so easily cast aside. But a linear narrative of abiogenesis would place the anthropic bottleneck at the origin of life, while the Stem Life narrative pushes it forward in time, into the realm of biological evolution.

## Conclusion

Cladistics provides a valuable conceptual tool for organizing thinking about origins, uniformly extending principles evidenced in organismal evolutionary history and the paleontological record. The proposed framework of “Stem Life” succinctly encapsulates these concepts and principles, and updates the pre-cladistics, ladderized view that often guides origins of life studies. This framework makes several predictions about the prebiotic world and the emergence of modern cellular life, with implications for interpreting prebiotic chemical experiments that seek to investigate these systems.

The vast majority of complex prebiotic systems with some features common to extant life would not be our direct ancestors. Geologically plausible experimental prebiotic chemical systems may be sampling this fecundity and, analogous to fossils representing stem groups, reveal subsets of characteristics shared with direct ancestors. As such, the lack of a plausible path or mechanism for transitioning from experimental prebiotic systems to extant biology is an invalid critique of the existence of the prebiotic system—it is only a valid critique of direct ancestry. Non-ancestral prebiotic systems could not merely be the by-product of the process of abiogenesis—the “failed experiments” on the path to life—but may in fact have been necessary for its eventual emergence, through providing a prebiosphere in which the earliest living systems could thrive.

Organic chemists may potentially discover several ways that complex prebiotic systems could emerge; even if there are good reasons to exclude these as likely precursors to life on Earth, such findings would substantially validate our expectations that abiogenesis is “robust” and that life has emerged elsewhere in the universe. Conversely, a series of linear transitions of increasing prebiotic complexity leading directly to celluar life and LUCA would not only be inconsistent with the processes of evolution observed for the rest of biology, but would violate a much broader Copernican Principle, having stark philosophical and astrobiological implications.
